# Design and validation of a tunable inertial microfluidic system for the efficient enrichment of circulating tumor cells in blood

**DOI:** 10.1002/btm2.10331

**Published:** 2022-04-29

**Authors:** Alejandro Rodríguez‐Pena, Estibaliz Armendariz, Alvaro Oyarbide, Xabier Morales, Sergio Ortiz‐Espinosa, Borja Ruiz‐Fernández de Córdoba, Denis Cochonneau, Iñaki Cornago, Dominique Heymann, Josepmaría Argemi, Delia D'Avola, Bruno Sangro, Fernando Lecanda, Ruben Pio, Iván Cortés‐Domínguez, Carlos Ortiz‐de‐Solórzano

**Affiliations:** ^1^ Program in Solid Tumors Center for Applied Medical Research (CIMA) Pamplona Spain; ^2^ Oncology Division Navarra's Health Research Institute (IDISNA) Pamplona Spain; ^3^ Automotive and Mechatronics R&D Foundation (Naitec) Pamplona Spain; ^4^ Department of Biochemistry and Genetics, School of Sciences University of Navarra Pamplona Spain; ^5^ Institut de Cancérologie de l'Ouest, “Tumor Heterogeneity and Precision Medicine” Lab., Blvd Jacques Monod Saint‐Herblain France; ^6^ Nantes Université, CNRS, US2B, UMR 6286 Nantes France; ^7^ Liver Unit, Clinica Universitaria de Navarra Pamplona (Navarra) Spain; ^8^ Centro de Investigación Biomédica en Red de Enfermedades hepáticas y Digestivas (CIBEREHD) Spain; ^9^ Centro de Investigación Biomédica en Red Cáncer (CIBERONC) Madrid Spain; ^10^ Department of Pathology, Anatomy and Physiology University of Navarra Pamplona Spain

**Keywords:** cell enrichment, cell sorting, circulating tumor cells, CTC, inertial forces, liquid biopsy

## Abstract

The analysis of circulating tumor cells (CTCs) in blood is a powerful noninvasive alternative to conventional tumor biopsy. Inertial‐based separation is a promising high‐throughput, marker‐free sorting strategy for the enrichment and isolation of CTCs. Here, we present and validate a double spiral microfluidic device that efficiently isolates CTCs with a fine‐tunable cut‐off value of 9 μm and a separation range of 2 μm. We designed the device based on computer simulations that introduce a novel, customized inertial force term, and provide practical fabrication guidelines. We validated the device using calibration beads, which allowed us to refine the simulations and redesign the device. Then we validated the redesigned device using blood samples and a murine model of metastatic breast cancer. Finally, as a proof of principle, we tested the device using peripheral blood from a patient with hepatocellular carcinoma, isolating more than 17 CTCs/ml, with purity/removal values of 96.03% and 99.99% of white blood cell and red blood cells, respectively. These results confirm highly efficient CTC isolation with a stringent cut‐off value and better separation results than the state of the art.

## 1 INTRODUCTION

Based on minimally invasive blood extraction, liquid biopsy can be used to stratify primary solid tumors and detect and characterize metastatic disease.^1^ This is based on the reasonable assumption that some characteristics of the primary tumor remain intact in tumor cells circulating in the bloodstream (CTCs). CTCs were first observed in 1869 by Ashworth.^2^ However, their first clinical uses had to wait until 2004, when the CellSearch® sytem^3^ (Menarini Silicon Biosystems, Bologna, Italy) was approved by the Food and Drug Administration (FDA). CellSearch® enumerates CTCs by detecting the expression of an epithelial cell surface marker (EpCAM), thus leaving undetected CTC subpopulations that do not express this marker, such as those that have undergone epithelial to mesenchymal transition (EMT), known to be important for critical steps of the metastatic cascade.^4^, ^5^, ^6^ Furthermore, CellSearch® fails to isolate CTCs from tumors in which EpCAM is rarely expressed, such as most squamous carcinomas, sarcomas, lymphomas, melanomas, or neurogenic tumors.^7^

Due to the limitations of the methods that, as CellSearch®, are based on cell surface marker specificity, other methods have been developed that rely on the detection of intrinsic properties of the CTCs.^8^ Parsortix® (ANGLE Biosciences, Toronto, Canada), for instance, enriches CTCs based on their size, by selecting cells able to deform as they traverse through small microchannels.^9^ Due to its promising results, ANGLE Biosciences has recently applied for FDA De Novo approval. Other methods, classified under the generic category of inertial sorting methods, take advantage of the balance between viscous, inertial, and secondary flow forces that cells undergo as they travel through microfluidic channels.^10^ Some of these methods have recently reached the market. In 2015, the ClearCell® FX1 System^11^ (Biolidics, Singapore) was launched, with the patented microfluidic biochip CTCChip® FR1. In 2017, Vortex Biosciences (Pleasanton, CA, USA) launched the VTX‐1® Liquid Biopsy System, a fully automated benchtop system for CTC isolation and collection.^12^ Both systems were successfully registered with the FDA as Class I Medical devices and obtained the European conformity (CE) marking for biomedical research. As drawbacks, ClearCell® FX1 requires blood lysis, thus compromising CTC viability, and both systems work with a fixed, relatively high cut‐off detection value of 13 or 14 μm, respectively.

Several inertial‐based systems, developed in academic environments, have been proposed to address these limitations.^13^ This is the case of the double spiral system,^14^ the contraction‐expansion array (CEA) microchannel,^15^ the spiral channel with periodic expansion structures,^16^ or the serpentine‐shaped microchannel.^17^ Other systems combine different techniques to obtain higher throughput and better performance.^18^, ^19^ Among these academic methods, the double spiral system provides a priori superior performance due to improved particle focusing and higher throughput. It was validated by separating 5 μm from 15 μm‐sized beads (i.e., 10 μm separation range), and two cancer cell lines (15.7 μm^20^ and 19.74 μm^21^ average cell sizes) from the remaining blood cell types, using a mixture of those cell lines with non‐lysed blood. Interesting as it is, this system lacks critical design and fabrication guidelines, including a design of the outlets that guarantee a balanced hydraulic resistance, required to ensure the appropriate particle trajectories toward their outlets. Furthermore, this system implements a high cut‐off separation value that might leave small CTCs undetected, and is yet to be evaluated in real CTC isolation scenarios.

In this article, we describe the design and fabrication of a double spiral inertial system, based on the background of the involved physical properties, computer simulations, and specific predefined separation requirements that improve the state of the art. We compared our computer simulations with experimental results obtained using calibration beads, which led to improved simulations that introduce a novel formulation of the lift coefficient of the inertial lift force term. This in turn allowed us to produce an improved, redesigned version of the device. Finally, we experimentally tested the CTC isolation capacity of our system in realistic scenarios consisting of cancer cell lines mixed in blood and with a mouse model of metastatic breast cancer, comparing the results obtained with a state‐of‐the‐art method. Then, as a proof of concept, a sample from a patient with hepatocellular carcinoma was processed to detect CTCs.

## 2 MATERIALS AND METHODS

### 2.1 Microfluidic chip design

As explained in the Background section (Supplementary Methods S1), the combination of inertial and Dean forces within a properly parametrized spiral‐shaped microfluidic channel can be used to separate particles based on their size (Figure S1). Several spiral designs have been proposed, for example, Fermat,^22^ Archimedean,^23^ or double Archimedean.^14^ These designs use trapezoidal^24^ or rectangular^25^ channel sections, and some use an extra inlet channel to focus all particles on one side of the channel at the beginning of the spiral.^26^ Among those, we chose a design based on a double Archimedean spiral due to its increased length compared to the simple spiral, which helps particle focusing. We aimed at developing a system able to separate particles with a cut‐off value of 9 μm, and a 2 μm separation range, that is, able to separate 8 μm from 10 μm particles, as would be required to separate CTCs larger than 9 μm, from erythrocytes and small leukocytes, without a significant loss of CTCs in the process. Besides this general goal, the specific requirements of our system, derived from the theory are: (#1) the system must focus particles into thin streamlines. This requires a minimum channel length (L_f_) that depends on the geometry of the channel, the diameter of the particle, and the fluid flow. Moreover, the system must satisfy the experimental value of confinement ratio (CR >0.07).^27^ (#2) The ratio between the lift and Dean forces within the channel must be close to 1. Besides these theory‐based requirements, an additional functional requirement was established (#3) to minimize the processing time, that is, maximize the flow speed.

Following Sun et al.,^14^ we opted for a double spiral channel with rectangular section, with 12 concentric loops that switch from counterclockwise to clockwise after the sixth loop (Figure S2a). Our device's cross‐sectional dimensions are 300 μm (width) and 85 μm (theoretical height). The total length of the device channel is 334 mm. The curvature radii (R) for each loop are listed in Figure S2a, with a constant valued distance of 450 μm between loops, rendering a minimum Dean value De of 6.94 at the exterior loop and a maximum De of 30.39 at the inner loop, for a flow of 860 μl/min that ensures a reasonably fast flow (requirement #3). With this geometry and considering the critical confinement ratio (CR = 0.07) and the channel height (H = 85 μm), positive values of confinement are guaranteed for particles larger than 5.95 μm (requirement #1). Furthermore, the device cross‐sectional dimensions render an aspect ratio (AR) of 0.283, which contributes to fulfilling requirement #1. Finally, the ratio between forces ranges from 0.5 for 6 μm particles to 5.5 for particles 20 μm in diameter. For our cut‐off value of 9 μm particles, the calculated ratio is 1.12. These ratios are close enough to one (requirement #2) to ensure sorting particles in the desired size range, that is, between 6 and 20 μm.

Special attention was paid to the design of the outlet section to ensure that each particle stream flows undisturbed into the desired outlet channel. A key parameter that affects the particle stream distribution is the resistance of the outlet, which is proportional to the flow, as described by Hagen‐Poiseuille's law.^28^ A poor definition of the outlet channel resistance may cause a streamline to be directed to a nondesired outlet, decreasing the efficiency of the system.^29^ Oh et al.^30^ established the need for balancing the pressure drop at all outlets that share a common point upstream. This condition, plus the fulfillment of Hagen‐Poiseuille's law, leads to the expression Q_1_S_1_ = Q_2_S_2_ = … = Q_N_S_N_ where Q_i_ is the flow at output i, and S_i_ is the section of the channel connected at i. This expression was used to calculate the flow required in each channel from the predefined resistance values. Furthermore, it is important to choose an appropriate tubing to avoid breaking the balance of output resistance. In our design, the outlet area consists of three output channels (Figure S2b): (i) the inner outlet has a section of 70 μm width by 85 μm height; (ii) the outer outlet has a section of 85 μm by 85 μm; (iii) and the middle outlet has a section of 145 μm by 85 μm.

Based on this design, one CAD file was produced (Figure S2d) from which two silicon wafers were fabricated (Wafers 1 and 2). The system was later redesigned to replace the output trifurcation (Figure S2b), with a simpler bifurcation, to widen the channel 66.6% (from 300 to 500 μm) before the bifurcation to allow easier particle separation (Figure S2c) and most importantly, to relocate the bifurcation, resulting in an outer channel of 412 μm in width by 85 μm in height and an inner channel of 103 μm by 85 μm, as suggested by new computer simulations that implement a novel approximation of the lift coefficient $\left(C_{L}\right)$ included in the expression of the inertial force (Supplementary Methods S4). Following this new design (Figure S2e), a new wafer (Wafer 3) was fabricated. Microfluidic devices were manufactured in polydimethylsilosxane (PDMS) via replica‐molding (Figures S2f and S2g) following the methodology described in Supplementary Methods S2.

## 3 RESULTS AND DISCUSSION

### 3.1 Optimization of the device: Fabrication protocol and linearity

The small area and long length of the microfluidic channel result in high hydraulic resistance, which in turn produces significant pressure loss within the device. The operating pressure needed to compensate for this loss, estimated at 4 bars for our target operating flow, can deform the PDMS, altering the geometry of the device. Therefore, we optimized the fabrication protocol to produce a rigid enough PDMS that would remain undeformed under this high pressure. For this purpose, inspired by Johnston et al.,^31^ we compared four PDMS curing protocols (Table 1).

**TABLE 1:** Fabrication protocol

Protocol	Curing temperature and time	PDMS: Curing
FP1	4 h at 70°C	10:1
FP2	4 h at 70°C + 16 h at 160°C	10:1
FP3	4 h at 70°C	8:1
FP4	4 h at 70°C + 16 h at 160°C	8:1

Five microfluidic devices were fabricated from Wafers 1 and 2 following each fabrication protocol. Five milliliters of water were inserted into the devices at increasing pressure values. For each device and pressure point, the average ratio between the experimental and theoretical flow (Q_exp_/Q_th_) was calculated (*n* = 5), to estimate the PDMS deformation.

**FIGURE 1:**
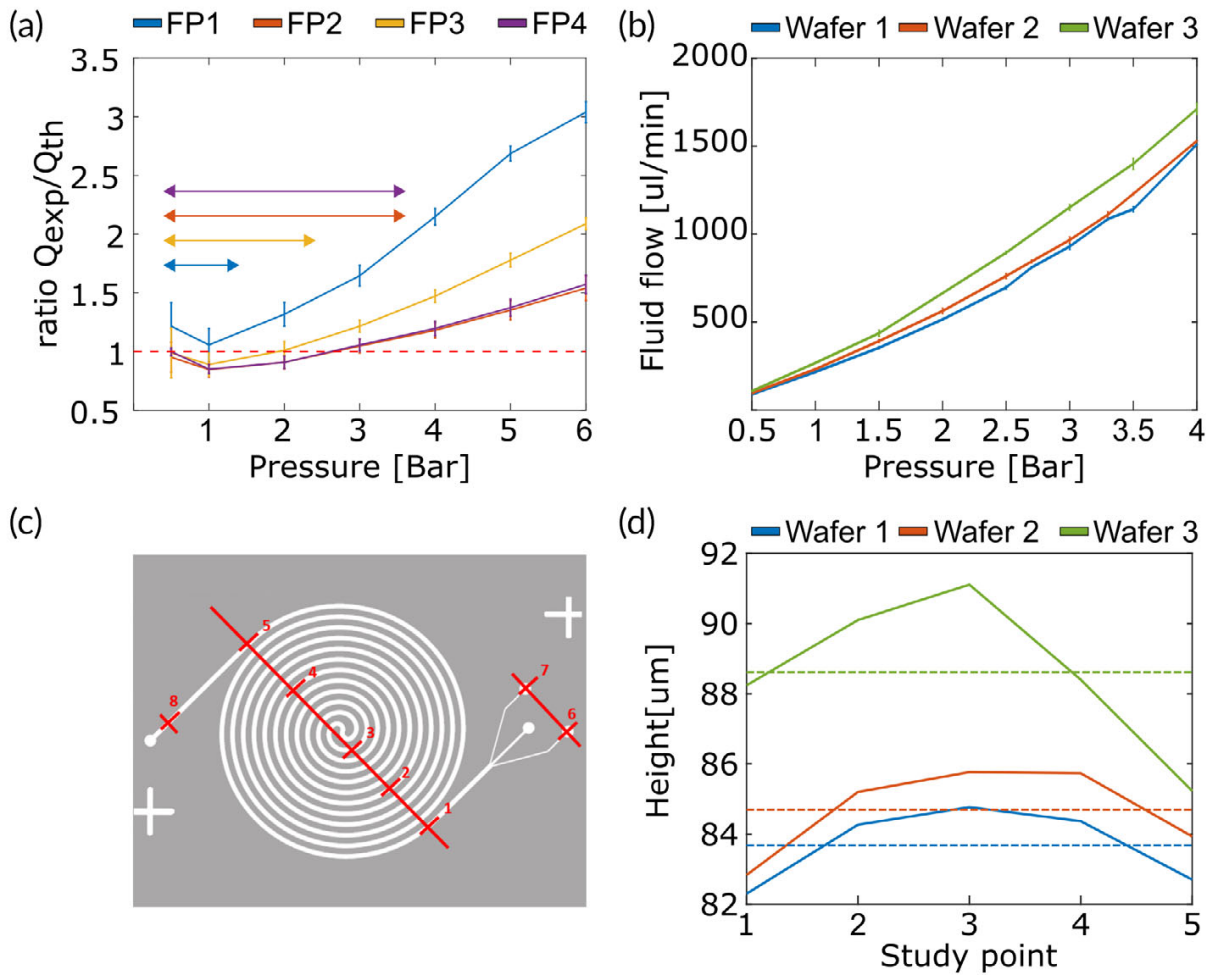
Polydimethylsilosane (PDMS) characterization study. (a) PDMS deformation for fabrication protocols FP1–FP4. The red dotted bar indicates the optimal $Q_{\exp}/Q_{\mathrm{th}}$ ratio of one, that is, perfectly rigid PDMS. Color arrowheads indicate the optimal, nondeformable working region for each fabrication protocol. (b) Relationship between flow and pressure for each wafer. (c) Height control points. (d) Height measurements and their average values (dotted lines) for the three wafers

The experimental flow Q_exp_ was obtained by measuring the volume extracted from the outlet system during 5 min using the setup described in Supplementary Methods S3. The theoretical flow Q_th_ was calculated from the geometry of the channel, thus assuming a perfectly rigid device. The results (Figure 1a) show that the protocols that use two temperatures and curation periods (FP2 and FP4) produce the most rigid microfluidic devices, as revealed by their lowest Q_exp_/Q_th_ ratios. Specifically, the ratios obtained using FP2 and FP4 are similar and remain below 1.05—that is 5% deformation—for working pressure values under 4 bars. Therefore, protocol FP2 was selected as it requires using less curing agent.

Next, we calculated the relationship between flow and pressure for the device. Five devices were fabricated from Wafers 1 to 3 following FP2. For each device, an average flow of 5 ml of water was measured experimentally at pressure points from 0.5 to 4 bar. As shown in Figure 1b, all devices display near‐linear behavior under most pressure regimes, except for the highest‐pressure values for which the PDMS starts to give way. Furthermore, higher flows were measured in Wafer 3 compared to Wafers 1 and 2, for the same pressure points. This can be explained by small differences in the height of the microchannels, confirmed from measurements taken using a DektakXT stylus profiler (Bruker, Billerica, Massachusetts, USA). To this end, eight points were measured in each device as shown in Figure 1c. The results shown in Figure 1d illustrate how the wafer fabrication protocol does not guarantee the theoretical height, as it slightly varies between wafers and within each wafer. This explains the differences seen in Figure 1b as, for a given pressure, devices built from Wafer 3 (from now referred to as wafer batch 1 redesigned or WB1r) exert lower resistance, thus allowing higher flows compared to Wafers 1 and 2, (from now on referred to indistinctly as wafer batch 1, or WB1 as they have identical design, and their channel height discrepancy is in average below 1%).

### 3.2 Optimization of the device: Computer simulations

Computer simulations (Supplementary Methods S4) were performed to obtain an accurate mathematical approximation of the behavior of the device for ideal, spherical particles. Especially critical was the implementation of a computational fluid dynamics (CFD) model of the net inertial lift force term $\;F_{L}$. This required crafting a lift force coefficient $\;(C_{L}$) for which no theoretical expression exists, and must be calculated from experimental data. Our initial simulation implemented al‐Amin's expression of $\left(C_{L}\right)$ ^22^ obtained by exponential fitting of Asmolov's experimental data (Cl=3.4368Re−0.714). Note that this expression does not take into account the channel geometry. WB1 wafers were fabricated under this assumption. Later, when our experimental results of bead trajectories for different flow rates were available for WB1, a reformulation of the CFD model was implemented based on the evaluation of different expressions of the lift coefficient $\;\;C_{l}$: we first used Zeng et al. experimental data,^32^ to approximate $C_{l}$ as 1.3665Re−0.59. Then, we reformulated the expression proposed by Zhou and Papautsky^27^ and converted it to a dimensionless expression that also incorporates the geometry of the device, allowing us to account for the channel height and its effect in the particle trajectories within the channel.

(1) Cl=ARH2WaRe=AR2HaRe

**FIGURE 2:**
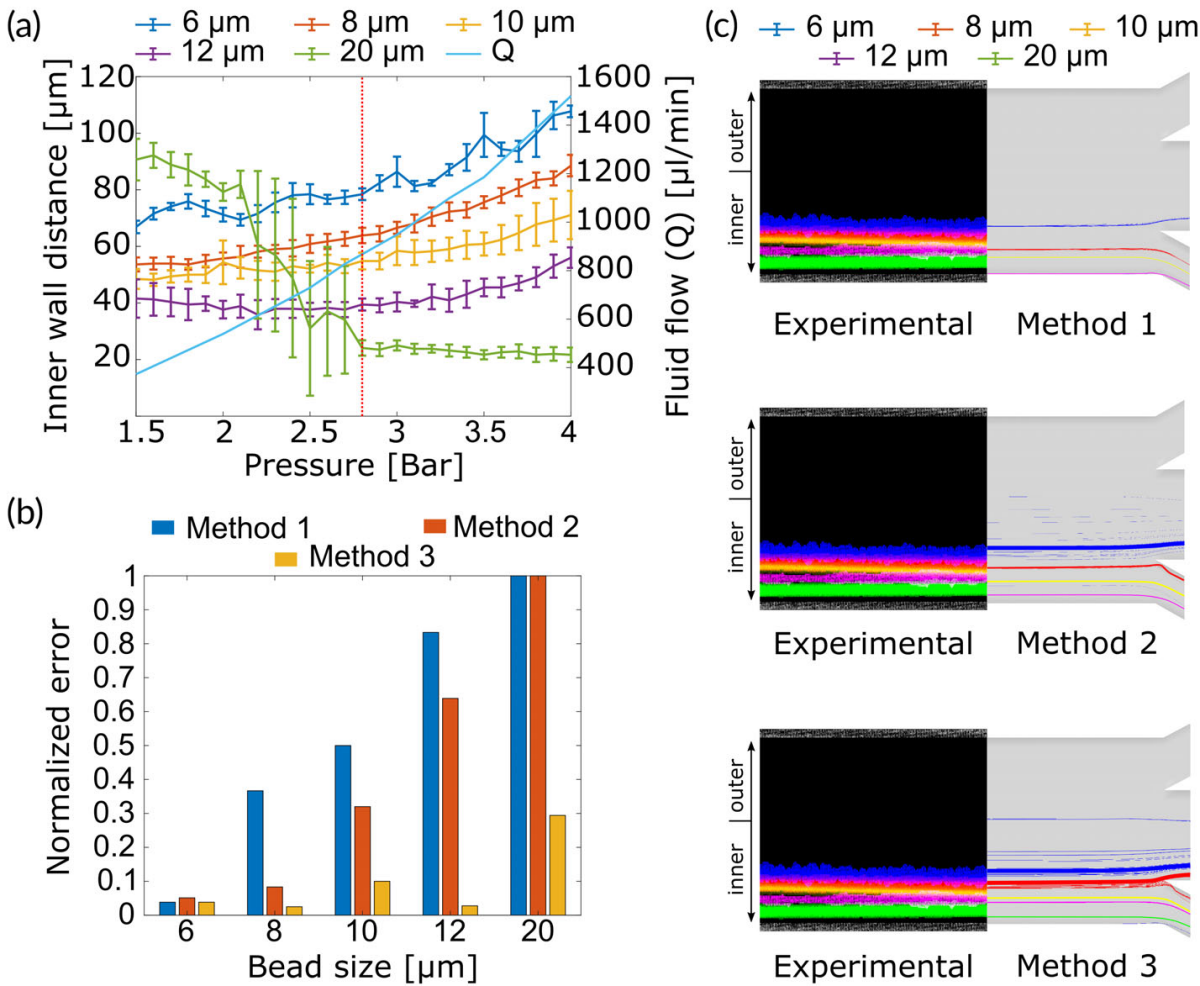
Experimental validation of our computer simulations. (a) Distance between the inner wall and bead streamlines for all bead populations, at increasing pressure regimes (wafer batch WB1). The vertical red dotted line indicates the working pressure corresponding to a flow of 860 μl/ml. (b) Normalized error between the location of the streamlines calculated experimentally, and the location indicated by our computer simulations, using three different computational approximations of the lift inertial force. (c) Visual comparison of the location of the bead streamlines calculated from the experimental results (left) and the computational simulations, using the three described approximations of the lift inertial force, measured at 860 μl/min. The experimental image was obtained by overlapping the fluorescence signal captured for each bead size analyzed in (a)

To evaluate these approximations of $C_{l}$, we compared the simulated and experimental distance between the bead streamlines and the inner wall, just upstream the outlet system. To this end, we used PMMA beads of 6, 8, 10, 12, and 20 μm (Table S1), prepared as described in Supplementary Methods S5. Three devices were fabricated using WB1. For each device and bead size, a solution of beads at 10^6^ beads/ml concentration was injected in the device at pressure values starting at 1500 mbar and ending at 4000 mbar, in 100 mbar steps. For each bead type and pressure point, we imaged the streamline at the end of the spiral and measured its distance to the inner channel wall (Supplementary Methods S6 and Figure S4). The average and standard deviation of the distance between each bead streamline and the inner side of the microchannel is shown in Figure 2a.

We then compared the experimental values obtained for WB1 with the simulated ones, for a working flow of 860 μl/min that corresponds to a pressure loss of 2800 mbar (see Figure 1b). Table S2 contains the experimental results, along with the theoretical values obtained from the computer simulations done using the three approximations of $C_{l}$. The normalized error between simulated (${\text{Dist}}_{\mathrm{sim}}$) and experimental (${\text{Dist}}_{\exp}$) was calculated as follows:

(2) $$\text{Normalized error}=\frac{\mathrm{abs}\left({\text{Dist}}_{\mathrm{sim}}-{\text{Dist}}_{\exp}\right)}{{\text{Dist}}_{\exp}}$$

Figure 2b shows that Al‐Amin's approximation of $C_{l}$ (Method #1) produces results that do not fit well with our experimental data. Our approximation based on Zheng et al. data (Method #2) reduces the error for small, 6, and 8 μm beads (0.1, 10%). However, the error remains high for larger beads. A major improvement was achieved by applying our proposed nondimensional version of Zhou and Papautsky's expression (Method #3), as the error was consistently lower compared to the other two approximations. Figure 2c illustrates these differences using artificially created images containing the streamlines obtained experimentally for all bead types, along with the computer‐simulated trajectories obtained using the three approximations of $C_{l}$. Consistent with these results, our new redesigned version WB1r was based on the simulations that implemented the last lift coefficient (Method #3), which could predict the stable trajectories for each bead size and helped us to define the location of the output bifurcation point to obtain a cut‐off value of 9 μm. Furthermore, using our simulations, we studied the effect of the channel height and working flow in the bead streamlines and therefore in the separation efficiency of the device (see Supplementary Methods S4).

### 3.3 Experimental validation of the device

**FIGURE 3:**
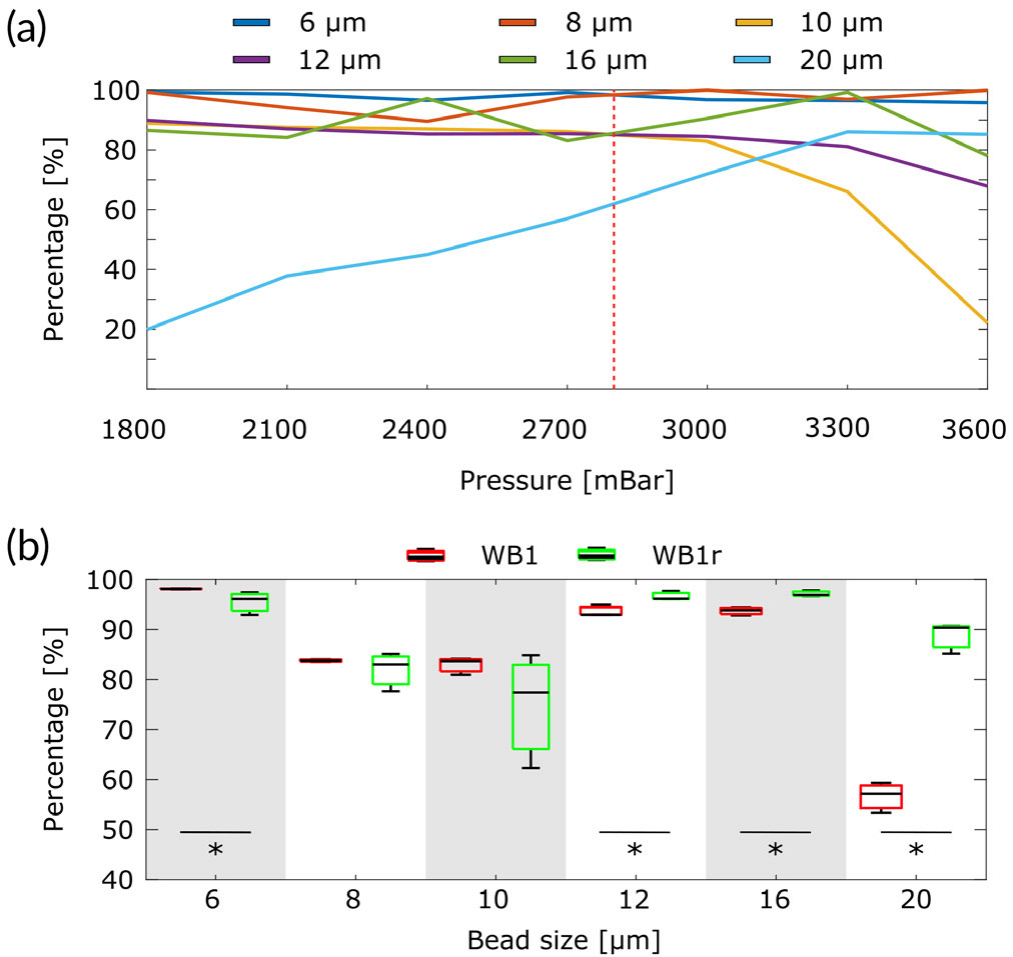
Validation of the inertial device using fluorescent calibration beads. (a) Yield of each bead population captured in its expected outlet (outer and middle outlet for 6 and 8 μm beads; and inner outlet for 10, 12, 16, and 20 μm beads) for increasing pressure working regimes. The vertical red dotted line shows the optimal working pressure (2800 mbar), corresponding to a flow of 860 μl/mL. (b) Yield values obtained for a mixture of all bead populations run through WB1 and WBr1 devices, used at their optimal working points. The experiment was repeated in three devices (*n* = 3). * Indicates statistically significant difference (*p* < 0.05) using the Mann–Whitney U test

We first quantified the enrichment capacity of our device using PMMA fluorescent beads of sizes 6, 8, 10, 12, 16, and 20 μm. The goal of this study was to evaluate the capacity of our device to separate beads based on their size, using a cut‐off value of 9 μm. To establish the optimal working point of the device, single‐type bead solutions were first run through a device fabricated using WB1, and the sorting capacity of the device was measured for increasing pressure settings of the microfluidic pump (1800, 2100, 2400, 2700, 3000, 3300, and 3600 mbar). For each bead type and pressure point, the outlets of the device were analyzed by flow cytometry. The yield for beads larger than 9 μm was calculated as the number of beads on the inner outlet divided by the total number of beads that were measured in the three outlets. The yield for beads smaller than 9 μm was calculated as the number of beads in the outer and middle outlets, divided by the total number of beads found in the three outlets. The results (Figure 3a) show that a working pressure of 2800 mbar guarantees the best separation efficiency, as the population of beads above the cut‐off value is mostly directed to the inner outlet, while the population below the cut‐off value is directed to the other two outlets. This optimal pressure working point corresponds to a velocity flow of 582 mm/s, calculated as V = Q/S, where Q (880 μl/min) can be obtained directly from its relationship with pressure (P) shown in Figure 1b and S is the section of the channel (300 μm × 84 μm). A similar experiment was performed for WB1r (300 μm × 89 μm), obtaining a working point value of 2600 mbar (940 μl/min), which corresponds to a velocity flow of 586 mm/s.

After the optimal working point was established, a sample containing a mixture of beads of all sizes was prepared with a final concentration of 2 × 10^5^ beads/ml. The sample was run through three devices (*n* = 3) fabricated from WB1, and WB1r. The content of the outlets was analyzed by flow cytometry to calculate the yield of each population. Our results (Figure 3b) show high separation efficiency for the original design (WB1) as all populations were properly collected in their expected outlets with more than 90% efficiency, except for the bead populations that are right below and above the cut‐off value—that is, 8 and 10 μm—for which the efficiency remained above 75%, and for the population of largest beads (20 μm) that were collected with an average efficiency of 60%. The performance for 8 and 10 μm beads can be explained by the highly demanding gap (2 μm), as interactions between particles may affect the separation process and the bead streamlines partly overlap. Regarding the results obtained for the largest, 20 μm size population, the relatively low‐performance measured could be due to the high inertial lift force that these particles suffer, which pushes them to remain near the middle of the channel. Globally, the yield efficiency of the device separating beads above and below 9 μm was 81.75%.

The results obtained with the redesigned version (WBr1, Figure 3b) confirmed the expected improvement, as the global efficiency of the device increased to 89.34 while suffering only a slight decrease in efficiency for the particles below the cutoff value. Altogether, we confirm for both designs a high efficiency while maintaining a very stringent separation gap of 2 μm around a demanding cut‐off value of 9 μm, which guarantees detection of a high percentage of CTCs. These values are in the order or larger than those obtained by other inertial systems, but very importantly, were obtained implementing a narrower validation gap of 2 μm. For instance, the original double spiral design by Sun et al.^14^ was tested separating beads by 5 μm versus 15 μm beads (i.e., with a resolution gap of 10 μm) and Gou et al.^16^ tested their design separating 6 and 10 μm versus 20 μm beads (i.e., with a resolution gap of 12 μm). Al‐halhouli et al.^33^ used a similar system to separate particles of 5 μm from particles of 10 and 15 μm, that is, with a resolution gap of 5 μm. They obtained a better resolution gap of 3 μm as they separated particles of 2 μm from particles of 5 and 10 μm.^34^ Finally, Mohamed et al.^35^ obtained also a separation gap of 3 μm, as they separated 7 and 10‐μm particles in a trifurcation where particles of 15 μm were sorted too, obtaining a final efficiency above 90% in all populations. These improved results are due to the use of a design that is based on specific requirements, simulated and optimized using a customized lift force coefficient; to the use of a balanced output system; and a PDMS fabrication protocol that guarantees very low channel deformation.

### 3.4 Mouse model of breast cancer metastasis

**FIGURE 4:**
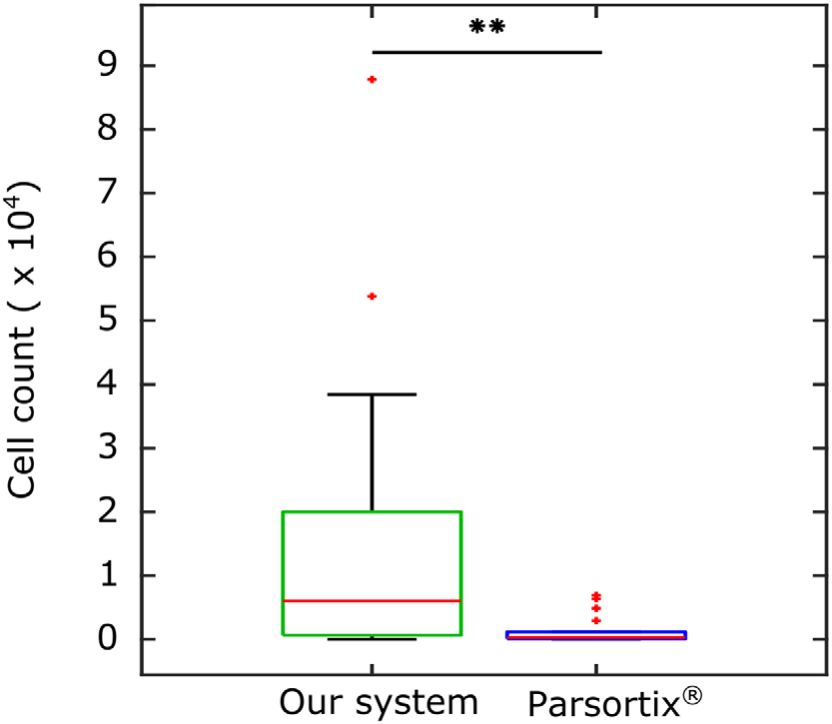
Comparison of our inertial system with Parsortix. Total number of CTCs detected per ml in all 18 mice. ** Indicates a statistically significant difference using a nonparametric Wilcoxon paired test (*p* < 0.01)

The ability of our device to detect and isolate CTCs from blood samples was validated in three steps: (i) first, we evaluated the effect of blood dilution on CTC detection efficiency, obtaining a threshold (1:94) below which the sorting efficiency drops considerably (Supplementary Methods S8 and Figure S5c); (ii) then we measured the effect of CTC concentration, confirming that the system efficiency does not depend on the concentration of CTCs, being on average 71.02% (Supplementary Methods S8 and Figure S5), and (iii) finally we used a murine metastasis model to validate our device in a more physiological scenario. In brief, 4T1‐GFP breast cancer cells were injected into the fat pad of the fourth right mammary gland of 18 mice. These cells develop primary tumors that are known to produce metastasis to the lungs. Blood samples were obtained from each animal at Day 21 and divided in two: (i) Sample #1, containing 50 μl of blood mixed with heparin and diluted in saline 1:200 (0.2% hematocrit) was processed the same day of extraction in our system (3400 mbar, 1150 μl/min); (ii) Sample #2, containing 740 μl of blood were analyzed by Parsortix® the following day. To that end, blood samples were kept in Cell‐Free DNA BCT® blood collection tubes (Streck, Nebraska, USA). Supplementary Table S3 shows the number of CTCs, per ml of blood, per mouse, detected by both methods. Figure 4 shows the total number of CTCs detected by both systems. As seen, our system provides higher detection efficiency (11‐fold) compared to Parsortix® (16,339 vs. 1412 cells/ml detected).

**FIGURE 5:**
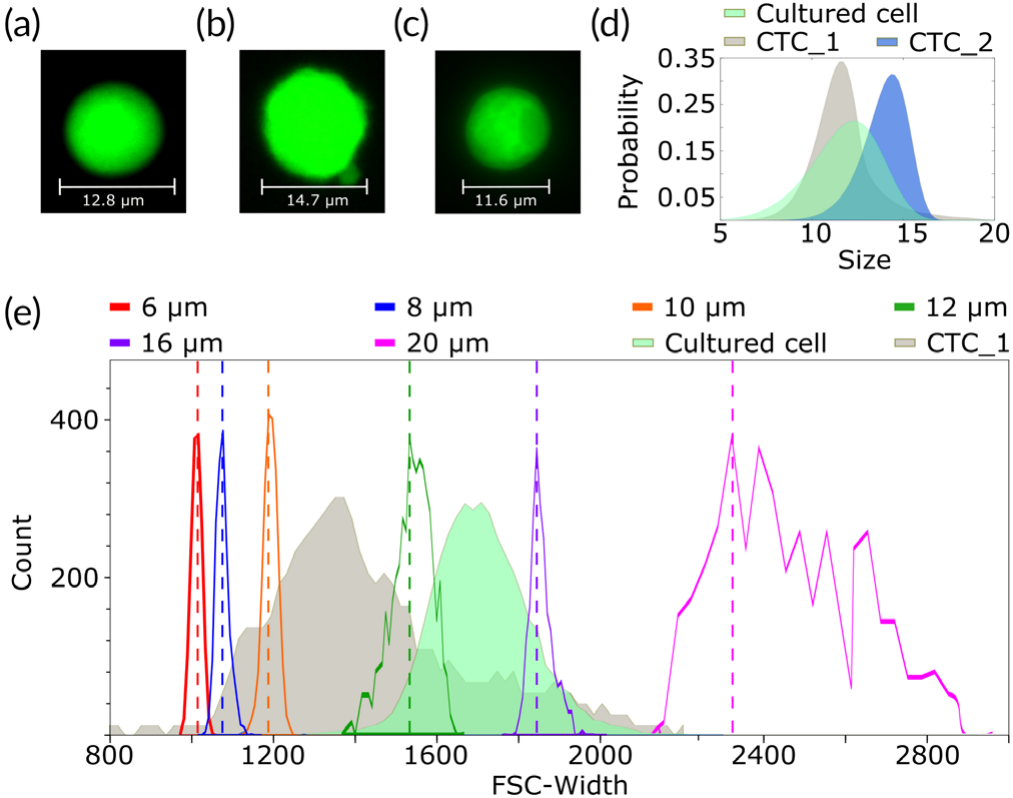
CTCs size analysis. (a) Sample cell from the original 4T1 cancer cell line imaged by fluorescent microscopy. (b) Sample CTC detected by Parsortix® imaged by fluorescent microscopy. (c) Sample CTC detected by our system imaged by fluorescent microscopy. (d) Size distribution of cells from the original cell line (Cultured cell), the CTCs detected by our system (CTC #1), and CTCs cells detected by Parsortix® (CTC #2). (e) Overlap of the width forward scattering parameter obtained by flow cytometry analysis for cultured cells (green) the CTCs detected by our inertial system (gray), and PMMA beads of different sizes

These differences in cell enrichment may be explained by the physical separation principle used by Parsortix®, which forces cells to deform through a 6.5 μm pore channel. Therefore, the nuclear size becomes the critical feature, which might lead to the detection of only the largest CTCs (Figure 5d). Regarding time performance, our system is 8.5% faster than Parsortix® for the same volume of processed blood. This difference is magnified considering that, in the same volume of blood, our system detects 11 times more CTCs than Parsortix®. Altogether, our system provides improved CTCs isolation with smaller sample volume and processing time. The reduced processing time should be in benefit of the cell viability for downstream analysis, and the small volume requirement might be beneficial in longitudinal studies that required multiple blood draws.

### 3.5 Morphological characterization of the detected CTCs

To characterize the detected CTCs, we measured their size from images taken using a fluorescence Zeiss Cell Observer® microscope with a 20× magnification objective and an Endow GFP spectral cube (#1031 346 Zeiss). Figure 5 shows a representative cell from the original 4T1 GFP cell line (Figure 5a), a cell detected with Parsortix® (Figure 5b), and a cell detected by our inertial system (Figure 5c). The probability distributions of cell sizes obtained from 141 cells of the original 4T1 GFP cell line (median value of 12.07 μm), 9 CTCs sorted by our system (median value of 11.25 μm), and 25 CTCs sorted by Parsortix® (median value of 14.29 μm) are shown in Figure 5d. These values prove that our method detects smaller CTCs than Parsortix® and interestingly, that the CTCs produced by the tumor are smaller than the original, injected cells. This confirms our requirement of using a cut‐off value of 9 μm to avoid missing the subpopulation made of small CTCs.

These differences found were confirmed by flow cytometry. Flow cytometry provides only relative size measurements. However, if the cytometer settings are maintained between acquisitions, relative differences between samples can be observed. Using this strategy, we performed a relative comparison of the sizes of isolated CTCs (Figure 5e, gray area) and the cultured cell population (Figure 5e, green area). To provide numerical references to these distributions, we plotted the forward scatter (FSC)—proportional to the particle size—of our mouse blood samples—isolated CTCs and cells from the 4T1 line—, with the FSC of single bead populations of sizes 6, 8, 10, 12, 16, and 20 μm. The results reveal that indeed, the detected CTC population, which is extracted from mice at Day 21, has a different phenotype from those injected at Day 0. Specifically, CTCs are 2 μm smaller on average than the original 4T1 cells. This difference is in agreement with that observed by other groups when quantifying the size of the CTCs population isolated from pancreatic tumors.^36^

### 3.6 Proof of concept with human samples

To calculate the purity of our sorted samples, we first measured the capacity of our system to remove the different blood subpopulations, by running blood from five healthy donors through our system. The working flow was established at 1150 μl/min. The sorted cells were labeled with CD45 and analyzed with flow cytometry. The obtained WBC and RBC counts (158.460 WBC/ml and 40.476 RBC/ml) were compared with the theoretical values (4 × 10^6^ WBC/ml and 5 × 10^9^ RBC/ml), obtaining average purity values of 96.03% and 99.99% removal, respectively. We then processed a blood sample taken from a patient with hepatocellular carcinoma in an advanced stage, carrying a highly vascularized tumor. The sample was processed in our microfluidic device immediately after blood extraction. Due to the large amount of blood to be processed (5 ml) and the required dilution (200 times in serum), a parallel configuration setup was used consisting in 12 devices (wb1r design) working in parallel at a total combined fluid flow of 13.8 ml/min. Therefore, the time needed to process 1 L of diluted blood was 73 min. During the entire process, the sample was kept on ice to help CTC preservation.

**FIGURE 6:**
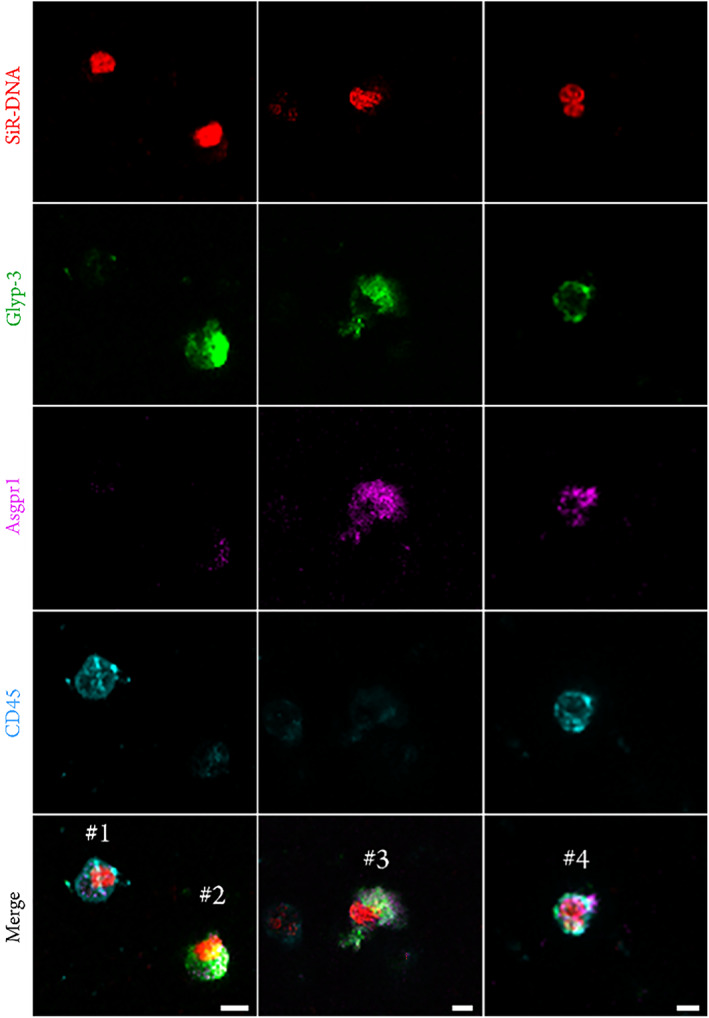
Cell surface profile isolated by our system. Each column shows a representative field of view of a microscope extension of isolated material. Cell #1 shows a representative cd45^+^ cell corresponding to the PBL subset. Cells #2 and #3 correspond to CTC candidate cells, which express glyp‐3^+^/asgpr1^−^/cd45^−^ and glyp‐3^+^/asgpr1^+^/cd45^−^ markers respectively. Cell #4 suggests a CHC cell profile (glyp‐3^+^/asgpr1^+^/CD45^+^). Scale bar: 5 μm

Cells isolated by our system were labeled with an antibody panel designed to identify CTCs (glyp‐3 and asgpr1), as well as the nuclear marker SiR‐DNA. Moreover, CD45 antibody was used to discard blood leucocytes (PBLs). Our confocal microscopy‐based analysis revealed at least 17 cells/ml, which show a glyp‐3^+^/asgpr1^−^/CD45^−^ (cell #2 in Figure 6) or glyp‐3^+^/asgpr1^+^/CD45^−^ (cell #3 in Figure 6) profile. These cells are candidates of being CTCs of hepatic origin. Another subset of cells displays a hybrid behavior (cell #4 in Figure 6), combining hematopoietic and tumor markers. According to the literature^37^ their presence has been associated with disease stage and overall survival.

## 4 CONCLUSIONS

Inertial sorting is an active area of investigation due to its potential for fast, marker‐free, size‐based detection and enrichment of CTCs. Several inertial sorting designs have been recently introduced, among which those based on simple or double spirals present some practical advantages. However, the design and implementation of these systems are weakly related to the physics that act within the devices. Furthermore, most of these devices have been only validated using artificial samples made of calibration beads or mixtures of blood with cancer cells. To address this, we have presented the design and optimization of a double spiral microfluidic device, using computer simulations that implement an improved version of the net inertial force term of the Navier–Stokes equation, to faithfully replicate the behavior of particles inside the device.

We have experimentally evaluated the relationship between the fluid velocity, the working pressure point, the cross‐section of the device, and the efficiency of the separation using calibration beads. We also provide an optimized fabrication protocol that ensures rigid enough PDMS for the pressure used. Then we validated the performance of the device using mouse blood artificially mixed with cancer cells, and a mouse model of cancer metastasis to test the performance of our system in a real scenario of CTC detection and enrichment. Finally, we performed a proof‐of‐concept experiment using human samples, obtaining an efficiency removing WBC and RBC of 96.03% and 99.99%, respectively, and isolating more than 17 candidate CTCs/ml from a hepatocellular carcinoma patient sample. In summary, in this article, we have presented and validated a double spiral microfluidic chip design that can sort and enrich CTCs from blood (starting at 1:94 dilution) at fluid flows ranged from 800 μl/min to 1.7 ml/min, which can be multiplied using parallel set‐ups to compensate for the dilution needed for larger samples, that is, 7 ml of whole blood. Our device displays a cut‐off value of 9 μm that can be modified by fine‐tuning the fluid flow or pressure applied to the system. Larger changes in the cut‐off value can also be performed using our computer simulations that implement an optimized version of the lift inertial force and incorporates the most relevant elements of the physics of the underlying forces. Table 2 summarizes the main differences between our system and a selection of the systems described in the literature, highlighting some of the advantages of our system. In summary, we believe that we have provided important theoretical, computational, and practical fabrication and experimental insights that prove the potential of inertial sorting as a tool for effective CTC isolation and enrichment.

**TABLE 2:** Comparison of the main CTC sorting devices described in the literature

		Our system	Parsortix	Sun et al.	Gou et al.	Al‐halhouli et al.		Yousuff et al.	Abdulla et al.
Validation: ComputerSimulation	Accuracy	>90% *except for 20 μm (70%)	–	>60%	Not quantified	–	–	78%, 95% and 82%	–
Validation: Beads	Diameter (μm)	6/8/10/12/16/20	–	5/15	6/10/20	5/10/15	2/5/10	7/10/15	5/8/15/24
	Resolution	2 μm	–	10 μm	10 μm	5 μm	3 μm	3 μm	3/7/9 μm
	Accuracy	>90% *except for 8–10 μm (80%)	–	>90%	82% (6) 88% (10) 99% (20)	–		>90%	94.8% (5) 80.8% (8) 75% (15) 84.4% (24)
Validation: Cells	Size (cell line)	12 μm (4 T1)	19.74 μm (MCF7)	15.7 μm (HeLa) 19.74 μm (MCF7)	15.7 μm (HeLa) 19.74 μm (MCF7)	–	–	–	15 μm (A549) 19.74 μm (MCF7)
	Yield	71%	78%	88.5%	89.5% and 93.5%	–	–	–	80.75% and 73%
Other validation	Murine models	Yes 11 folds CTCs/ml than Parsortix	Yes	No	No	No	No	No	No
General features	Cut‐off	9 μm (tunable)	6.5 μm	Between 5 and 15	Between 10 and 20	Between 5 and 10	Between 2 and 5	Multiple	Between 8 and 15
	Sample	Diluted in serum	Whole blood	Diluted in serum	Diluted in PBS	–	–	–	Lysed or diluted
	Removed cells	96.03% WBC 99.99% RBC	99% WBC 100% RBC	92.28% BC	99% RBC	–	–	–	–
	Channel shape	Double spiral	Stepped height	Double spiral	CEA spiral	Spiral	Spiral	Spiral	Combine serpentine and spiral
	Fluid flow	1.4 ml/min (tunable)	83 μl/min	333 μl/min	750 μl/min	3 ml/min	600 μl/min	1.8 ml/min	1.8 ml/min
Ref #			9	14	16	33	34	35	38

## AUTHOR CONTRIBUTIONS

**Alejandro Rodríguez‐Pena:** Conceptualization (equal); data curation (equal); investigation (equal); methodology (equal); software (equal); validation (equal); writing – original draft (equal). **Estibaliz Armendariz:** Funding acquisition (equal); methodology (equal); project administration (equal); software (lead). **Alvaro Oyarbide:** Investigation (equal). **Xabier Morales:** Investigation (equal); writing – original draft (supporting). **Sergio Ortiz‐Espinosa:** Investigation (equal). **Borja Ruiz‐Fernández de Córdoba:** Investigation (equal). **Denis Cochonneau:** Investigation (equal). **Iñaki Cornago:** Methodology (supporting). **Dominique Heymann:** Resources (equal). **Josepmaría Argemi:** Methodology (supporting); resources (equal). **Delia D'avola:** Methodology (supporting); resources (equal). **Bruno Sangro:** Methodology (supporting); resources (equal). **Fernando Lecanda:** Funding acquisition (equal); resources (equal). **Ruben Pio:** Funding acquisition (equal); resources (equal). **Iván Cortés‐Domínguez:** Conceptualization (equal); funding acquisition (equal); methodology (equal); project administration (equal); resources (equal); writing – original draft (equal). **Carlos Ortiz‐de‐Solórzano:** Conceptualization (equal); funding acquisition (equal); methodology (equal); project administration (equal); resources (equal); writing – original draft (equal).

### PEER REVIEW

The peer review history for this article is available at https://publons.com/publon/10.1002/btm2.10331.

## Supporting information


Appendix S1 Supporting Information

## Data Availability

The data that support the findings of this study are available from the corresponding author upon reasonable request.
