# Supplementary material for: Design and validation of a tunable inertial microfluidic system for the efficient enrichment of circulating tumor cells in blood
Source: Bioeng Transl Med. 2022 Apr 29;7(3):e10331. doi: 10.1002/btm2.10331 (PMC9472016; doi:10.1002/btm2.10331)
Supplement: Supplementary file 1 — Appendix S1 Supporting Information [file BTM2-7-e10331-s001.pdf]

Bioengineering & Translational Medicine

SUPPORTING INFORMATION

Design and validation of a tunable inertial microfluidic system for the efficient enrichment of circulating tumor cells in blood

Alejandro Rodríguez-Pena[a,b], Estibaliz Armendariz[c], Alvaro Oyarbide[a,b], Xabier Morales[a,b], Sergio Ortiz-Espinosa[a,b,d], Borja Ruiz-Fernández de Córdoba[b], Denis Cochonneau[e], Iñaki Cornago[c], Dominique Heymann[e,f], Josepmaría Argemi[g,h], Delia D'Avola[g,h], Bruno Sangro[g,h] , Fernando Lecanda[a,b,i,j], Ruben Pio[a,b,d,i], Iván Cortés-Domínguez[†,a,b] and Carlos Ortiz-de-Solórzano[†,*,a,b,i]

Content

Supplementary Methods S1. Background 2
Supplementary Methods S2. Fabrication 4
Supplementary Methods S3 Experimental setup 4
Supplementary Methods S4. Computer simulations 5
Supplementary Methods S5. Preparation of beads 7
Supplementary Methods S6. Image analysis 8
Supplementary Methods S7. Cells and Cell Culture 8
Supplementary Methods S8. Mouse model 8
Supplementary Tables 11
Supplementary Figures 12
Supplementary Bibliography 15

Supplementary Methods S1. Background

The principal forces that act on suspended Newtonian particles are briefly described in the following paragraphs:

Viscous drag force.

A particle immersed in a fluid suffers a force F_{Drag} that opposes its movement. For a spherical particle:

$$F_{Drag}=S \cdot f_{Drag}=\frac{\pi a^2}{4} \cdot f_{Drag} \quad (1)$$

where S is the cross-sectional area [m^2] of the particle, a is the diameter of the particle [m] and f_{Drag} is a coefficient that can adopt four different expressions depending on the value of $R_p = Re(a/H)^2$, being Re the Reynolds number of the flow and H the channel height[1]. In most practical situations, R_p< 0.2 and this coefficient adopts the following form:

$$f_{Drag} = 12\frac{\mu v_t}{a} \quad (2)$$

being v_t the velocity of the fluid relative to the particle.

Inertial lift forces

A flowing Newtonian particle migrates laterally until reaching an equilibrium position, the location of which depends on the cross-sectional shape of the channel. In circular-shaped channels, the equilibrium position consists of an annulus located 0.6 times the channel radius. In square-shaped channels, four equilibrium positions exist located in the middle of each side. Finally, if the cross-section is rectangular, two equilibrium positions exist, located towards the center of the long sides of the rectangle (Supplementary Figure 1a)[2].

To migrate towards their equilibrium position, particles suffer inertial lift forces (F_L)[3]. The theory about inertial lift forces was developed in the 1990s by Asmolov et *al.*, who expressed F_L as a function of a scaling factor, i.e. $F_L \propto \rho U_m^2 a^4/D_H^2$ where U_m is the mean fluid velocity and D_H is the hydraulic diameter of the channel[4]. Recent studies by Di Carlo et *al.*[2] distinguish two forces: a *shear-gradient lift force* (F_S) that acts near the centerline of the channel and depends on a scaling factor $F_S \propto \rho U_m^2 a^3/D_H$, and a *wall lift force* (F_W) that acts near the walls of the channel and depends on a scaling factor $F_W \propto \rho U_m^2 a^6/D_H^4$. However, it is common practice to work with the expression proposed by Asmolov, known as *net inertial lift force*, dependent on a non-dimensional lift coefficient (C_l), i.e.:

$$F_L = C_l\frac{\rho U_m^2 a^4}{D_H^2} \quad (3)$$

Using Asmolov's data[4], Al Amin[5] empirically fitted the lift coefficient C_l to an asymptotic expression dependent only on the Reynolds number $C_{l=}$ $(3.4368\ Re^{-0.714})$. More recently, Zhou and Papautsky[6] reformulated this coefficient, incorporating also the effect of the diameter of the

particle and the geometry of the channel: $C_L \propto H^2/(a\sqrt{Re}), W > H$, where H and W are the height and width of the channel respectively. Using this approximation, the authors also calculated the minimum channel length required for flowing particles to reach the equilibrium positions, L_f:

$$L_f = \frac{3\pi\mu D_h^2 L_m}{2\rho U_f C_l a^3} \quad (4a)$$

where L_m is the lateral migration distance and U_f the mean fluid velocity. Alternately, Di Carlo et *al.*[2] derived their estimate of the minimum channel length:

$$L_f = \frac{\pi\mu W^2}{\rho U_m C_l a^2} \quad (4b)$$

where U_m is the maximum fluid flow ($U_m = 2U_f$). In practice, both approximations tend to underestimate the distance, so it is common practice to overestimate the length of the channel to ensure that all particles reach the equilibrium position. Interestingly, as the aspect ratio (AR) of the channel increases, the equilibrium positions become delocalized, producing less usable broader regions[7], as it has been modeled and shown experimentally[8].

Dean drag force.

Inertial migration forces have only been used recently for particle sorting, due to the complexity caused by the existence of multiple equilibrium positions. To overcome this problem, novel methods make use of the centripetal force that particles undergo as they change their direction of motion within a curved channel. This so-called Dean force reduces the pressure on the outer side of the channel, by generating a pressure gradient towards that side, thus moving the particles in that direction. This outward movement, in turn, pushes the fluid located near the outer side of the channel towards the inner side, via both the bottom and top of the channel, generating two symmetrical vortices known as Dean's vortices (Supplementary Figure 1b). Due to this secondary Dean flow, all equilibrium positions produced by the inertial lift forces become unstable and two new horizontally symmetrical equilibrium positions appear near the inner side of the channel (Supplementary Figure 1b), where an equilibrium between all involved forces is reached. These Dean forces have already been used in designs based on spiral[9] or serpentine[10,11] shaped-channels. The Dean flow was first experimentally demonstrated in 1910[12]. Later, in 1927, W. R. Dean provided a theoretical description of this phenomenon[13] and related it to a dimensionless Dean number (De). Berger et *al.*[14] provided a generally accepted form of this number, and related it to the hydraulic diameter of the channel (D_H):

$$De = \sqrt[2]{\frac{D_H}{2R}} \cdot Re \quad (5)$$

The Dean force (F_D) is normally expressed as $F_D \sim \rho U_m^2 a D_H^2 / R$, where R is the radius of curvature of the curved channel. In practice, it is important to emphasize that the equilibrium positions are located at a distance from the wall that increases with *De*[15] until reaching a threshold value that should be avoided, above which the particles are dragged towards the center of the channel by the Dean vortex. A recent study based on computer simulations[16] confirms these experimental observations first obtained by Martel and Toner[17], who highlighted that the transition through the Dean vortex occurs more easily as the particle size decreases, due to the strong dependence of the shear-gradient lift force with the particle size. During this transition, particles can show different focus modes based on the so-called particle confinement ratio $CR = a/H$ that has been experimentally studied in curved channels with low aspect-ratio[18], distinguishing three modes. When CR is larger than a critical value, experimentally set to 0.07, the particles align in a narrow stream (*focus mode*). If CR is lower than 0.07, the particles enter a wider particle stream (*rough focus mode*). For even lower CR values, particles spread along the whole channel width focus is not achieved (*unfocused mode*). Furthermore, numerical simulations, showed that the center of the main vortex is located in the inner side of the channel for low values of *De* and moves towards the outer sides of the channel as *De* increases, creating a secondary Dean Vortex. Nivedita et *al.*[19] experimentally and numerically demonstrated that the cut-off *De* value at which the secondary Dean flow is formed decreases linearly with AR.

Supplementary Methods S2. Fabrication

Microfluidic devices were fabricated on Sylgard 184 Polydimethylsiloxane (PDMS) (Dow Corning Co., Midland, USA) via replica-molding, using patterned 100 mm diameter circular silicon wafer molds. Wafers 1 and 2 were fabricated by the Scottish Microelectronics Centre, The University of Edinburgh, UK, and wafer 3 by Lionix BV (The Netherlands) by deep reactive ion etching (DRIE) technology. To fabricate the microfluidic devices, PDMS was mixed 1:10 with the curing agent (1 part of curing agent by ten parts of PDMS), poured into the silicon wafer, degassed and heated for 4 hours at 70ºC. The polymerized PDMS was then unmolded and inlet/outlet holes were created using biopsy punches (Electron Microscopy Sciences, Hatfield, PA, USA) of 0.75 mm diameter for wafer 1-2 and 1.5 mm diameter for wafer 3. The patterned PDMS was irreversibly attached to a regular glass coverslip by oxygen plasma bonding at 0.3 mBar for 90 seconds in a Zepto system (Electronic Diener, Ebhausen, Germany). After covalent bonding, the device was heated again for 16 hours at 160 ºC to increase PDMS rigidity.

Supplementary Methods S3 Experimental setup

Each microfluidic device is connected to a tubing system for sample insertion and withdrawal (Supplementary Figure 3a). The outlet system for wafers 1-2 consists of three 40 cm long 30G Polytetrafluoroethylene (PTFE) tubes (Cole-palmer, Illinois, USA). This setup guarantees that 15.5% of the sample goes to the inner outlet, 62.8 % to the middle outlet, and 21.6 % to the outer

outlet (data measured experimentally). The outlet for wafer 3 consists of two 40 cm long 20G PTFE tubes. The inlet system for wafer 1-2 consists of a 53 cm long 30 G PTFE tube, inserted in a 10 cm length 24G PTFE tube which acts as a reducer for a 23 cm long 20G PTFE tube that fits in a hermetic pressurized cap. The inlet for wafer 3 consists of a 50 cm long 20G PTFE tube inserted in the inlet. To insert the sample in the device we used an XL LineUp FlowEZ pressure pump (Fluigent, Okabé, France) connected to a 15 ml Falcon tube with a characteristic air connector p-cap adaptor to 20G tubing (Supplementary Figure 3c), thus providing a hermetic environment. The outlet PTFE tubes are inserted in a 3D holding piece, printed *in house*, which holds a stepper motor that automatically moves the PTFE tubes to their unloading positions (Supplementary Figure 3b). The stepper motor and the attached holding piece is controlled using an Arduino-based system, ChipKit, (Diligent Corporation, New York, USA) designed to provide reliable, repetitive extraction of the sample from the microfluidic device outputs. The ChipKit controls a 5V CC stepper motor (Shenzhen AOKEYU Technology Co, Shenzhen, China) attached to a 3D printed holding a piece that positions the outlet PTFE tubes above their respective repositories one at a time. An LCD shows the operation mode (see next paragraph) and the time countdown.

Three working modes were programmed. The first mode or *volume measure mode*, positions all three-outlet PTFE tubes simultaneously into the same unloading position during a predefined time. This allows measuring the output flow as the ratio between the total volume unloaded divided by the unloading time. The second mode, *individual recovery mode*, moves each outlet to a different unloading position that contains a different cytometry tube. As in the first mode, the unloading time can be predefined. The third*, manual mode*, is the same as the second one except for the time parameter, which is removed, allowing manual operation.

Supplementary Methods S4. Computer simulations

Numerical approach

Numerical simulations were used to evaluate how factors such as the design geometry, fluid properties, or the operating regime affect the dynamics of particles within the flowing fluid.

Our double spiral geometry was modeled ANSYS Workbench v19. The ANSYS Design Modeler module was used to generate and parametrize the spiral geometry. ANSYS Meshing module was used to create the 3D mesh. ANSYS Fluent was used as solver and postprocessing tool. Modeling was based on a two-component Lagrange discrete phase model: fluid, treated as a continuum phase, and particles treated as a dispersed phase.

The continuum phase -i.e., fluid- was solved using the Navier-Stokes equation[20] assuming a steady-state flow:

$$\rho(\vec{v} \cdot \nabla)\vec{v} = -\nabla P + \mu\nabla^2\vec{v} + \vec{\gamma}\rho \qquad (6)$$

where the inertial term (left side of Equation 1) contains a convective term that has no time dependency. The right side of the equation contains a Pressure Gradient ($-\nabla \mathrm{P}$), a Diffusion Term ($\mu\nabla^2\vec{v}$) and ($\vec{\gamma}\rho$), which represents any non-extended body force. ρ is the density of the fluid

[kg/m^3], P is the pressure [Pa] and μ is the dynamic viscosity [Pa · s]. Particle trajectories were solved integrating the force balance on Newton's second law of motion:

$$\frac{d\vec{v}_p}{dt} = \frac{18\mu C_D Re_s}{\rho_p a^2 24}(\vec{v} - \vec{v}_p) + \frac{\vec{g}(\rho_p - \rho)}{\rho_p} + \frac{1}{2}\frac{\rho}{\rho_p}\frac{d(\vec{v} - \vec{v}_p)}{dt} + \frac{F_L}{\frac{1}{6}\pi a^3 \rho_p} \quad (7)$$

where $\vec{v}_p$ and $\vec{v}$ are particle and flow velocity vectors, $\vec{g}$ is the gravitational acceleration, μ is fluid viscosity, C_D is the drag coefficient defined by Morsi and Alexander[21], ρ_p and ρ are particle and fluid densities, a is particle diameter and t is time. On the right side of Equation 2, the first term is the drag force per unit mass, the second term is the force caused by gravity per unit mass and the third term is the virtual mass force per unit mass to accelerate the fluid surrounding the particle. These three terms are implemented in ANSYS Fluent. The fourth and last term is the net inertial lift force per unit mass derived by Asmolov (see **ESI-S1**). This term is not implemented in ANSYS, and was introduced in the CFD model via a User Defined Function (UDF)[22] compiled with Visual Studio 2017.

Equations (6) and (7) are "two-way coupled", i.e. the flow field affects the particles' trajectories and the particles' properties can, in turn, alter the flow field. Flow equations were solved using a second-order upwind scheme with the SIMPLEC algorithm for pressure-velocity coupling. The discrete phase (particle trajectories) was solved using 106 time-steps in a tracking technology known as Direct Particle Method. The boundary conditions of the model were kept as simple as possible: a velocity inlet boundary condition was applied at the channel inlet to match the flow rates first described in the bibliography and later confirmed by our experimental results. All outlets were set at atmospheric pressure. On the walls, a no-slip condition was applied. All particles could escape the domain at inlet and outlet boundaries and at walls collisions and reflections were considered perfectly elastic, with a restitution coefficient of 1. Interactions between particles were neglected and all particles were considered inert to maintain computing time at a manageable scale. The working fluid material properties were those of water at 25ºC, and the injected particles represented spherical PMMA beads with various diameter sizes: 6, 8, 10, 12, 16, and 20 µm. All bead populations were uniformly distributed at the inlet.

Channel height dependency in the particle location

As explained in the Results section of the manuscript, we reformulated the expression proposed by Zhou and Papautsky[6] to approach the lift coefficient, as using it we could simulate our experiments with small errors. This nondimensional expression takes into account the channel geometry, among other factors, and in particular the height and the width. This way, we can indirectly analyze the influence of the height of the device in its particle separation performance, by analyzing its effect in the particle trajectories. To this end computer simulations were performed using three device heights: 80, 84 and 89 µm. Correction coefficients were applied to heights to account for the channels' volume increase due to high working pressure (3000 – 3200mBar). Based on the results presented in Figure 1 of the manuscript, the correction factor

used was 1.05 (i.e., 5% deformation). Therefore the actual channel heights simulated were 84, 88 and 93 µm. We simulated these devices at two working flow regimes: 860 µl/min (the one used in our validation experiments, with a channel height -deformation corrected- of H = 88µm), and 1000 µl/min. The evaluation of the height and the comparison with the experimental values is shown in the following table:

	CFD Results						Experimental	
Device Height H	84 (80+4)		88 (84+4)		93 (89+4)		88 (84+4)	
Q [µl/min]	860	1000	860	1000	860	1000	860	1000
Bead Ø [µm]	Distance from the inner Wall [µm]							
6	73.5	77	81	83	83	87	78	82
8	55	58	61.5	63	63	65	60	64
10	42	46	45	55	48.5	57	50	56

Effect of the device height. Distance between the inner wall and the bead streamlines for 6, 8 and 10 µm beads, using three different channel heights at two different fluid flows.

As shown in this table, the CFD model shows the best agreement with the experimental results using height H = 88 (80+4) µm. Also, the table illustrates the height dependency and its effect in the beads' distance from the inner wall. As the device height increases, the distance from the inner wall ¡ increases for all bead sizes, as expected from the bibliography[15]. These results indirectly show how the performance of the device is affected by errors in the height caused by fabrication errors, as they show the deviation of the cut-off value from the intended one. In brief, an increase in the height of the device causes an increase in the effective cut-off value. Alternatively, before the device is fabricated, our results show where the output channel bifurcation must be located, given the intended channel height and cut-off value. Finally, the cut-off value can also be fine-tuned by adjusting the working flow regime, as shown by the variation of trajectories caused by the flow. This is illustrated in the table above by showing the differences in the particle trajectories at flows of 860 µl/min vs 1000 µl/min.

Supplementary Methods S5. Preparation of beads

Polymethyl methacrylate (PMMA) fluorescence microbeads (PolyAn, Berlin, Germany) of different sizes and colors, were used for validation purposes.

To prevent adhesion and the formation of clusters of beads, Tween20 was added to the mixture. Supplementary table 3 shows the characteristics of each bead population. For a specific bead diameter (Ø), the volume used (V) to obtain the desired concentration of beads (C) in 10 ml of DI water was calculated as:

$$V = \frac{1{,}19 * \pi * 100}{6} * C * \emptyset^3 \qquad (8)$$

Supplementary Methods S6. Image analysis

Twenty-six (26) greyscale images were acquired per sample using a monochromatic camera (Coolsnap, Teledyne Photometrics, Arizona, USA) attached to an Axiovert 200 fluorescence microscope (ZEISS, Oberkochen, Germany) with a 2,5X objective lens focused at the end of the spiral channel. Concretely, the sample was excited through the appropriate spectral cubes: DAPI (#488049 Zeiss) for blue beads, Endow GFP (#1031-346 Zeiss) for green beads, and Rhodamine (#488020 Zeiss) for red and pink beads.

To measure the separation between the streamline and the device wall, a three-step MATLAB algorithm was implemented to analyze the images (Supplementary Figure 4a). First, the images were automatically rotated to ensure that the end of the spiral was vertically aligned. To this end, we applied the Canny edge detector algorithm to binarize the lower half of the image, which corresponds to the straight part of the channel, before the trifurcation. The output was analyzed using the Hough Transform to detect the angle of inclination of the channel walls. This angle was applied to transform the images using a simple rotation transformation. The resulting rotated images were automatically cropped to include only the straight section of the microchannel, located just before the trifurcation, as shown in Supplementary Figure 4b. To this end, the bead streamline position was calculated by measuring the mean value of each pixel column, and once located, the image was cropped allowing 180 pixels at each side of the bead streamline position, to ensure that the entire width of the channel remains inside the cropped image (Supplementary Figure 4b). Then, the algorithm locates the edges of the microchannel, calculated as the minimum mean value in each side of the bead streamline (blue and yellow narrow lines in Supplementary Figure 4c and Supplementary Figure 4d). Finally, the distance of the bead streamline from the inner wall channel was measured from the known pixel size (1,81 µm).

Supplementary Methods S7. Cells and Cell Culture

4T1-GFP cells were cultured in RPMI 1640 medium supplemented with 10% fetal bovine serum and 1% antibiotic (penicillin-streptomycin solution) at 37ºC and 5 % CO_2 atmosphere. Every forty-eight hours cells were trypsinized and subcultured in culture flasks to maintain a conducive environment for cell proliferation. 24 hours before an experiment, cells were kept in sub-confluence to avoid cluster formation.

Supplementary Methods S8. Mouse model

All animal experiments were housed under pathogen-free conditions at center for applied medical research (registration number ES31-2010000132) and conducted following protocols approved by the local Institutional Animal Care Committee (protocols 113-13 and 049-18).

To study the effect of the dilution of the blood in the CTC detection efficiency,non-lysed blood extracted from 3 mice was mixed with saline solution at six concentrations (1:6, 1:31, 1:62, 1:94,

1:187, and 1:250), simulating hematocrit values of 6.66%, 1.29%, 0.64%, 0.32%, 0.21%, and 0.16%. Then $3x10^4$ cells of the breast cancer 4T1-GFP breast cancer cell line (see Supplementary Methods S7) were mixed with 5 ml of blood resulting in a final cell concentration of $6x10^3$ cells/ml per sample. Samples were run through the inertial system (WB1) for 1 minute at 3400 mBar -i.e.,1150 µl/min flow-. This increased flow was required to compensate for the higher density and viscosity of the sample, as well as the deformability of cells. The material directed to each outlet of the device was stored, and the inner outlet sample, expected to contain the CTCs was analyzed by flow cytometry using a two-step gating strategy: first the GFP- population was eliminated by plotting the fluorescence intensity (B525-FITC-A) against the side scatter area (SSC-A), which is related to the cell complexity (Supplementary Figure 5a). Then, for the remaining population (GFP+), single cells were selected by plotting SSC-A against the side scatter height (SSC-H) and choosing the elements located in the diagonal area of the plot (Supplementary Figure 5b). Our results show an efficiency threshold at dilution 1:94 (0.42% hematocrit) (Supplementary Figure 5c) below which the sorting efficiency of the system drops considerably. This could be explained since, at low dilution, cells suffer more cell-cell interactions during their itinerary towards the equilibrium flowing positions, causing less focused trajectories and poor inertial focusing which causes some cancer cells to be directed to the wrong outlet.

To study the robustness of the system at isolating CTCs in a controlled way -i.e., based on a comparison with a ground truth sample containing a known number of CTCs-. To this end, we prepared two groups of samples: (i) Group #1, consisted of 7 samples of saline mixed with 4T1 cancer cells at concentrations of 25, 100, 500, 2000, 6000, and $5x10^4$ cells/ml; (ii) and Group #2, made of 7 samples with whole mouse blood diluted 1:200 dilution in saline (0.2% hematocrit) mixed with 4T1 cells at the same concentrations used in Group #1.

The concentration of each sample was confirmed by flow cytometry. For Group #1 samples, 1 ml of the sample was run through the inertial device at 3400 mBar (1150 µl/min flow) and the device outlets were analyzed by flow cytometry. Supplementary Figure 5d shows the average yield (n=3), measured as the percentage of cancer cells that enter the device and are detected in their expected inner outlet. As shown, the results provide an average efficiency of 71.02%. An honest Tukey significant difference test confirms that system performance is not dependent on CTCs concentration. On the contrary, regarding Group #2 isolation performance, the overwhelming presence of erythrocytes in the middle outlet makes it impossible to detect by flow cytometry the small number of cancer cells in that outlet. Therefore, instead of using the yield, we compared the number of cancer cells detected in the inner channel with those detected in Group #1 -i.e., using saline-, in optimal quantifiable conditions. Results in Supplementary Figure 5e show similar cell detection values for all concentrations tested except for the lowest one -i.e., 10 cells/ml-. This could be explained by methodological errors. However, no significant differences were observed either with the medium -i.e., saline or blood- or with the level of cell concentration. These results highlight that the expected performance of the system would not differ from the values obtained

with saline medium. The results also show a 4.58x enrichment factor, calculated by dividing the average efficiency (71.02%) by the percentage of the fluid volume that is redirected towards the inner outlet (15.5% of the initial volume). Other systems have been validated using cells but were tuned to separate largely different cell types, or required prior lysis. This is the case of the system presented by Syed et al.[23], which separates two different microalgae cell populations of 6 and 10 µm suspended on a marine medium, obtaining a sorting efficiency of 90%, or Sun et al.[24] who separate larger cancer cells -i.e., HELA and MCF-7 - mixed in blood, from the remaining blood cell types obtaining an efficiency of 88.5%. A more complex system was developed and validated by Abdulla et al.[25] who processed blood samples mixed with two tumor cell lines –i.e., A549 and MCF-7-. Their results revealed the need for lysing the sample to obtain a good sorting efficiency of the tumor cell lines (above 73%), similar to the efficiency that we obtain without the need for lysing the blood sample. This is important since, in a real CTC detection scenario, lysis is normally avoided as it could affect the viability of the CTCs. Our alternative to avoid lysing is to dilute the blood to reduce the number of cells that pass through the system at a given time, as done by Gou et al 14 who obtained a high efficiency –i.e., more than 93.5%-, isolating MCF-7 cells from 100x diluted blood at 750 µl/min. It should be noted that we validated our system using cells of much smaller size than the MCF-7, which explains the lower efficiency obtained.

To produce a model of cancer metastasis in which validate the performance of our system detecting CTCs, 4T1-GPF cells (2 x 10^4) were inoculated in the fat pad of the 4th right mammary gland of 18 female c-Rag2 Balb/c mice between 8 and 12 weeks old. Blood (850 µl) was extracted from the heart before the euthanasia of the animals by using a 25G needle connected to a 1 ml syringe (SS+01H25161, TERUMO, Tokyo, Japan).

Supplementary Tables

PMMA Beads characteristics						
Nominal	6	8	10	12	16	20
Size (SD) [µm]	5.92 (0.31)	8.02 (0.45)	9.87 (0.48)	12.12 (0.71)	16.01 (0.74)	20.23 (1.31)
Excitation [nm]	510 to 580	350 to 400	510 to 580	415 to 480	350 To 400	450 to 565
Emission [nm]	570 to 630	400 To 480	570 to 630	470 to 550	400 to 480	540 to 620

Supplementary table 1. PMMA bead characteristics

Bead Ø [µm]	Distance from inner wall [µm]			
	Method #1	Method #2	Method #3	Experimental
6	75	82	81	78
8	38	55	61.5	60
10	25	34	45	50
12	6	13	35	36
20	0	0	12	17

Supplementary table 2. Validation of computer simulations. Distance between the inner wall and the bead streamlines for all bead populations, at the optimal working point of wafer batch WB1, using three different computational approximations of the lift inertial force: Method #1, Al Amin's approximation based on Asmolov data[5]; Method #2, our approximation based on Zeng et *al.*[24]; and Method #3, our approximation reformulating the term proposed by Zhou and Papautsky[19]. For theoretical calculations, an increment of 5% was applied in the height of the channel (H=84+4 µm).

**Estimation of CTCs/ml**									
**Mouse #**	1	2	3	4	5	6	7	8	9
**Our system**	6,435	26,087	478	20,000	19,683	174	0	1,391	87,855
**Parsortix**	1,162	0	213	723	4	790	6	4,865	122
**Mouse #**	10	11	12	13	14	15	16	17	18
**Our system**	5,623	53,797	17,910	8,996	2,261	3,826	638	522	38,428
**Parsortix**	699	6,370	0	35	364	74	2,916	161	6,919

Supplementary table 3 Comparison with Pasortix®. Comparison of the number of CTCs detected by our system and the commercial Parsortix® system from blood samples isolated from the breast cancer model.

Supplementary Figures

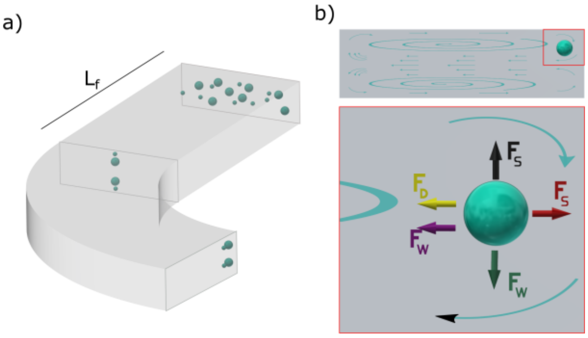

Supplementary Figure 1. Equilibrium positions are caused by inertial lift forces. (a) For a rectangular channel, where the aspect ratio (AR) differs from 1, the stable positions are reduced to two, located in the middle of the long faces of the cross-section, due to the flatness of the velocity profile in the large dimension. Lf is the minimum distance for placing all the particles in stable positions. In curved channels, those stable positions are moved through the inner wall. (b) Representation of the secondary Dean flow in curved rectangular channels and the forces that are involved –i.e., Shear lift force (F_S) in both vertical and horizontal component (black and red arrows); wall lift force (F_W) in both vertical and horizontal component (green and purple arrows) and the dean force (F_D) represented in the horizontal component with a yellow arrow-

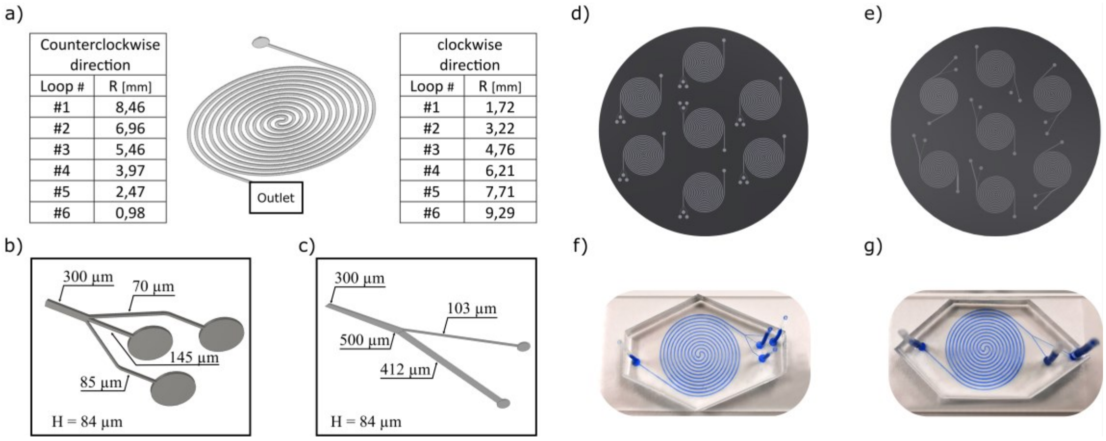

Supplementary Figure 2. Concept and design of our double Archimedean spiral system. (a) Conceptual design and characteristic dimensions. (b) Outlet design (initial version). (c) Outlet design (redesigned version). (d) CAD of wafer 1-2. (e) CAD of wafer 3. (f) Microfluidic device fabricated from wafer 1. (g) Microfluidic device fabricated from wafer 3.

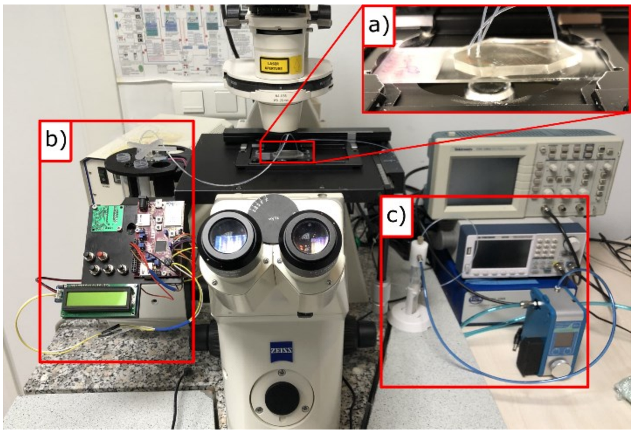

Supplementary Figure 3. Set-up of the system. (a) Microfluidic device, (b) output controlling system, and (c) pumping system.

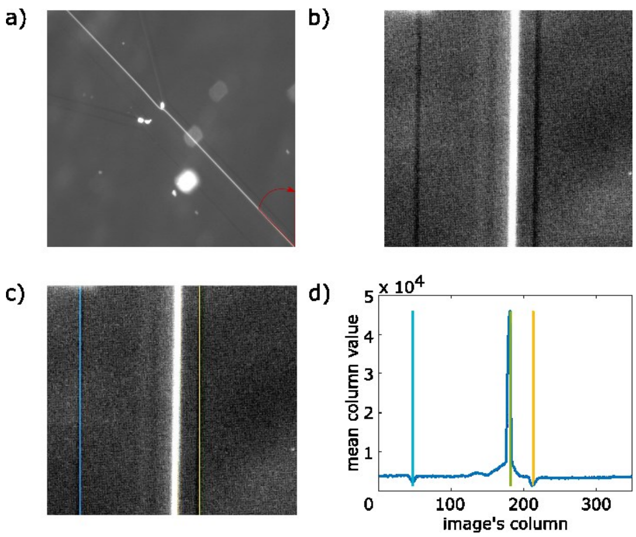

Supplementary Figure 4. Example of the algorithm that calculates the location of the bead streamline. (a) Raw image. (b) Preprocessed image after cropping and rotating the raw image. (c) Overlap the preprocessed image and the detected left-edge position (blue line) and right-edge position of the microchannel. (d) A plot of the mean column pixels value showing the position of the beads (green line).

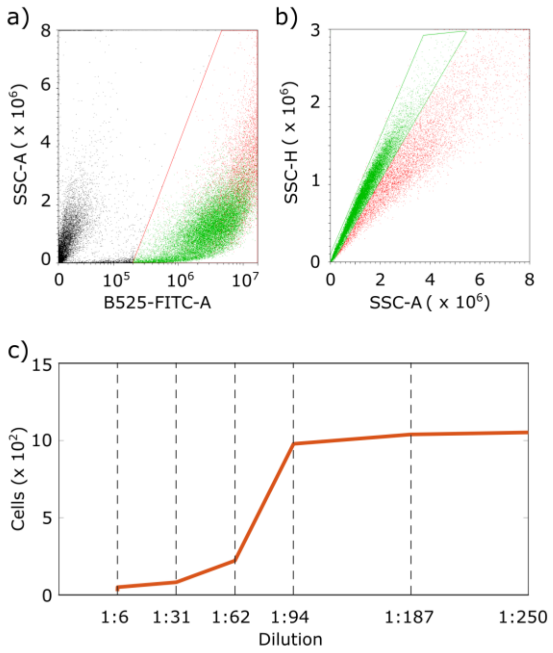

Supplementary Figure 5. System validation: effect of dilution and concentration on the detection of 4T1 cancer cells mixed in blood samples and its comparison with the commercial platform Parsortix®. (a) Representative flow cytometry gating strategy used to select the population of GFP+ cells (n=3). (b) Representative flow cytometry gating strategy used to select single GFP+ cells. (c) Number of tumor cells detected by flow cytometry in the outlet of the system, for increasing dilution levels (n=3). (d) Recovery rate of cancer cells suspended in saline solution, as a function of the initial concentration of cancer cells. (e) Concentration of cancer cells recovered in saline solution (Blue boxes), and non-lysed blood (Red boxes), as a function of the initial concentration (n=3). The x-axis represents the averaged initial concentrations. No statistically significant differences were found either in the performance of the device or in the medium and concentration levels using Tukey's honest significant test, t-test, and Mann-Whitney U test (p>0.05), respectively.

Supplementary Bibliography

1. Zhang J, Yan S, Yuan D, et al. Fundamentals and applications of inertial microfluidics: a review. *Lab Chip*. 2016;16(1):10-34. doi:10.1039/C5LC01159K
2. Di Carlo D. Inertial microfluidics. *Lab on a Chip*. 2009;9(21):3038-3046. doi:10.1039/b912547g
3. Matas JP, Morris JF, Guazzelli E. Lateral forces on a sphere. *Oil and Gas Science and Technology*. 2004;59(1):59-70. doi:10.2516/ogst:2004006
4. Asmolov ES. The inertial lift on a spherical particle in a plane poiseuille flow at large channel Reynolds number. *Journal of Fluid Mechanics*. 1999;381:63-87. doi:10.1017/S0022112098003474
5. Amin A Al. High Throughput Particle Separation Using Differential Fermat Spiral. Published online 2014.
6. Zhou J, Papautsky I. Fundamentals of inertial focusing in microchannels. *Lab on a Chip*. 2013;13(6):1121. doi:10.1039/c2lc41248a
7. Reece AE, Oakeya J. Long-range forces affecting equilibrium inertial focusing behavior in straight high aspect ratio microfluidic channels. *Physics of Fluids*. 2016;28(4):043303. doi:10.1063/1.4946829
8. Gupta A, Chow L, Kumar R, Ladd AJC. Effect of aspect ratio on inertial migration of neutrally buoyant spheres in a rectangular channel. In: *47th AIAA Aerospace Sciences Meeting Including the New Horizons Forum and Aerospace Exposition*. ; 2009:1022. doi:10.2514/6.2009-1022
9. Sun J, Liu C, Li M, et al. Size-based hydrodynamic rare tumor cell separation in curved microfluidic channels. *Biomicrofluidics*. 2013;7(1). doi:10.1063/1.4774311
10. Ying Y, Lin Y. Inertial Focusing and Separation of Particles in Similar Curved Channels. *Scientific Reports*. 2019;9(1):1-12. doi:10.1038/s41598-019-52983-z
11. Özbey A, Karimzadehkhouei M, Akgönül S, Gozuacik D, Koşar A. Inertial focusing of microparticles in curvilinear microchannels. *Scientific Reports*. 2016;6(1):1-11. doi:10.1038/srep38809
12. Flow of water in curved pipes. *Proceedings of the Royal Society of London Series A, Containing Papers of a Mathematical and Physical Character*. 1910;84(568):107-118. doi:10.1098/rspa.1910.0061
13. Dean WR. XVI. Note on the motion of fluid in a curved pipe . *The London, Edinburgh, and Dublin Philosophical Magazine and Journal of Science*. 1927;4(20):208-223. doi:10.1080/14786440708564324
14. Berger SA, Talbot L, Yao LS. Flow in Curved Pipes. *Annual Review of Fluid Mechanics*. 1983;15:461-512. doi:10.1146/annurev.fl.15.010183.002333
15. Kuntaegowdanahalli SS, Bhagat AAS, Kumar G, Papautsky I. Inertial microfluidics for continuous particle separation in spiral microchannels. *Lab on a Chip*. 2009;9(20):2973-2980. doi:10.1039/b908271a
16. Garcia M, Pennathur S. A model for inertial particles in curvilinear flows. *Microfluidics and Nanofluidics*. 2019;23(5):63. doi:10.1007/s10404-019-2234-x
17. Martel JM, Toner M. Particle focusing in curved microfluidic channels. *Scientific Reports*. 2013;3:1-8. doi:10.1038/srep03340
18. Xiang N, Shi Z, Tang W, Huang D, Zhang X, Ni Z. Improved understanding of particle migration modes in spiral inertial microfluidic devices. *RSC Advances*. 2015;5(94):77264-77273. doi:10.1039/c5ra13292d

19. Nivedita N, Ligrani P, Papautsky I. Dean Flow Dynamics in Low-Aspect Ratio Spiral Microchannels. *Scientific Reports*. 2017;7:1-10. doi:10.1038/srep44072
20. Batchelor GK. *An Introduction to Fluid Dynamics*.; 2000. doi:10.1017/cbo9780511800955
21. Morsi SA, Alexander AJ. An investigation of particle trajectories in two-phase flow systems. *Journal of Fluid Mechanics*. 1972;55(2):193-208. doi:10.1017/S0022112072001806
22. Ansys Inc. ANSYS Fluent UDF Manual. *Knowledge Creation Diffusion Utilization*. 2013;15317(November):724-746.
23. Syed MS, Rafeie M, Vandamme D, et al. Selective separation of microalgae cells using inertial microfluidics. *Bioresource Technology*. 2018;252:91-99. doi:10.1016/j.biortech.2017.12.065
24. Sun J, Li M, Liu C, et al. Double spiral microchannel for label-free tumor cell separation and enrichment. *Lab on a chip*. 2012;12(20):3952-3960. doi:10.1039/c2lc40679a
25. Abdulla A, Liu W, Gholamipour-Shirazi A, Sun J, Ding X. High-Throughput Isolation of Circulating Tumor Cells Using Cascaded Inertial Focusing Microfluidic Channel. *Analytical Chemistry*. 2018;90(7):4397-4405. doi:10.1021/acs.analchem.7b04210
